# Targeting the Complement C1 Proteases Using Camelid-Derived Single Domain Antibodies: Production, Characterization and Possible Application for Tissue/Fluid Analysis

**DOI:** 10.3390/antib15050092

**Published:** 2026-10-05

**Authors:** Nolwenn Haut, Véronique Rossi, Julie Peliconi, Jean-Baptiste Reiser, Nicole M. Thielens, Lubka T. Roumenina, Christine Gaboriaud

**Affiliations:** 1 University Grenoble Alpes, CNRS, CEA, IBS, Team CIRDis, F-38000 Grenoble, France; nolwenn.haut@ibs.fr (N.H.);; 2 Centre de Recherche des Cordeliers, Team Inflammation, Complement and Cancer, Institut National de la Santé et de la Recherche Médicale, Sorbonne Université, Université de Paris Cité, 15 Rue de l’Ecole de Medecine, F-75006 Paris, France; 3 University Hospital Federation (FHU) COMET, F-75006 Paris, France

**Keywords:** classical complement pathway, nanobody, serine proteases, subcellular localization

## Abstract

Background. The complement C1r and C1s proteases initiate the classical complement pathway within the C1 complex, but emerging evidence points to additional roles in tissues, including cell-intrinsic functions linked to cancer and connective tissue disorders. Few tools are available to explore these plasma and tissue-relevant functions. We report the first development and characterization of anti-C1r and anti-C1s VHH single-domain antibodies (nanobodies). Methods. A lama was immunized with the purified human C1r2/C1s2 tetramer. After phage display selection, two anti-C1r VHHs (C1rCl1, C1rCl6) and one anti-C1s VHH (C1sCl7) were produced in *E. coli* and characterized by ELISA, Western blot, SPR and size-exclusion chromatography using recombinant protease fragments and proenzyme forms. Functional impact on C1s activation was assessed by cleavage assay, and tissue applicability by immunohistochemistry on human skin sections. Results. All three VHHs bound with high affinity to both active and proenzyme forms. Epitope mapping localized C1rCl1 and C1rCl6 to the CCP2 module of C1r, and C1sCl7 to the CCP1 module of C1s. The two anti-C1r VHHs recognize distinct, non-overlapping epitopes and can bind simultaneously to C1r. C1rCl1 mildly inhibited C1s activation by C1r. The VHH pair C1rCl1/C1rCl6 enabled sandwich ELISA detection of C1r in serum, plasma, and cell culture supernatant. Immunohistochemistry on human skin confirmed clean C1r staining in keratinocytes and fibroblasts, with a dissociated tissue distribution compared to C1s. Conclusions. These first anti-C1r and anti-C1s nanobodies constitute useful tools for detection in biological fluids and tissue staining, with potential applications in diseases where these proteases play a pathological role.

## 1. Introduction

The complement proteases C1r and C1s are emerging as versatile effectors operating within the well-established complement cascade, with recently discovered functions inside cells and tissues that may be independent of the C1 complex [1]. Fully characterizing these properties requires well-validated detection tools capable of recognizing both the plasma-derived and cell-intrinsic forms of these proteases in their native and pathological biological context, including in connective tissue diseases, autoimmunity and cancer.

The complement components C1r and C1s were initially discovered as the proteases initiating the classical complement cascade, as part of the C1 complex, where they are associated with the C1q recognition protein [2]. While C1q is produced by the tissue resident macrophages, C1r and C1s are mainly produced by the liver hepatocytes but also by fibroblasts and, in cancer, the tumor cells [3,4]. C1r and C1s are closely related at the gene level on chromosome 12 and predominantly found within the C1 complex in the circulation [5]. The classical complement pathway is mainly activated when the C1 complex binds to immune complexes. This recognition event triggers the proteolytic activation of C1r, which then activates C1s. The two proteases are indeed secreted in a proenzyme form, and they get activated on site (where C1q binds) through the cleavage of a highly specific Arg-Ile peptide bond, known as the activation site. This cleavage triggers a conformational change towards the active form within the terminal serine protease domain [6,7].

Activation of the classical complement pathway is involved in pathogen elimination and in host homeostasis. As such, it plays a defensive and protective role at the interface between innate and acquired immunity. One major exception is when the classical complement pathway is activated by autoantibodies targeting host cells or tissues, leading to their accelerated destruction. This is the case for cold agglutinin disease, a rare autoimmune hemolytic anemia, which has led to the development and approval of the inhibiting anti-C1s sutimlimab antibody as a therapeutic agent [8,9].

Pathogenic genetic variants of C1R or C1S are very rare and are either associated with autoimmunity, in the context of systemic lupus erythematosus (when the two alleles are affected) or to a dominant gain of function mutation in the case of periodontal Ehlers-Danlos Syndrome (pEDS) [10,11,12]. One link with connective tissue abnormalities in pEDS is an alternative function of complement C1s in the degradation of collagen I [13]. Another cleavage target of C1s recently identified is the alarmin HMGB1, potentially modulating its inflammatory signal [14]. On the other hand, C1r complement serine protease was shown to contribute to kidney fibrosis [15]. Dysregulation may also come from hypermethylation and downregulation (e.g., advanced metabolic dysfunction-associated steatotic liver disease) [16].

In the specific context of tumor progression, the complement system works as a double edge sword, its impact deeply varying depending on the environment, i.e., the type of cancer [17,18,19]. C1r and C1s are at best studied in clear cell renal cell carcinoma (ccRCC), where their overexpression is associated with poor prognosis [17]. In this context C1r and C1s are expressed by the cancer-associated fibroblasts and tumor cells and associate with tumor-associated macrophages derived C1q to activate complement within the tumor microenvironment [4,20]. Tumor cell-secreted C1r is a driver of metastasis by promoting anoikis resistance through upregulation of ITGB1 (integrin beta-1)-mediated Akt/Erk signaling and inhibition of the Fas/FasL apoptotic pathway [21]. Finally, intracellular C1s (and likely C1r) in tumor cells, regulate cell proliferation and the crosstalk with the tumor-infiltrating T cells [20]. Cell-intrinsic functions of C1r and C1s promoting tumor growth and poor prognosis are not limited to renal cancer [20], but are shared with cutaneous squamous cell carcinoma [22] and oral squamous cell carcinoma [23]. However, highly expressed C1r and C1s are of good prognostic for triple negative breast cancer, mainly due to cell-intrinsic regulation of chemokines secretion and cytotoxic T cell recruitment [24].

Despite this expanding landscape of biological relevance, the field has limited molecular tools to study C1s and particularly C1r. New tools are necessary to localize these complement components within tissues, distinguish their individual contributions, and probe their intracellular functions. Conventional polyclonal antibodies offer limited specificity, and no single-domain antibody targeting C1r or C1s has been reported.

Mono-domain antibodies (VHH), also called nanobodies, are specifically derived from the heavy chain of camelids antibodies [25]. They are small (15 kDa), robust and easy to produce in prokaryotic systems. Interestingly, they can provide new tools for diagnostic, research and therapeutics, since they are highly soluble, thermostable, show high affinity and low immunogenicity, as well as excellent tissue penetration, among general valuable properties.

Here we present the first anti-C1r and anti-C1s VHH, the strategy used to generate them, their main characteristics, as well as their utility for biological sample analyses.

## 2. Materials and Methods

### 2.1. Generation of C1r and C1s VHHs

The generation of the VHHs had been outsourced to Nabgen company (Marseille, France). The animal experiment protocol was approved before its application on the 25 April 2022 by the Ethics Committee on the French minister APAFIS platform, referenced as APAFIS #36263-2022033115403140 v2. Briefly, a lama (*Lama glama*) that had not been vaccinated for at least six months was immunized with three injections of 0.77 mg of human purified and reassembled (C1r)_2_/(C1s)_2_ tetramer prepared as described below. After blood collection, isolation of lymphocytes was followed by reverse transcription to synthetize cDNA from total RNA. The cDNA was used for PCR amplification to obtain the sequence coding for the variable’s domains of heavy-chain antibodies. A VHH phage display library was obtained by cloning the PCR amplicons into phagemid pHEN4 vector. The selection and the screening of the VHHs was performed by two rounds of panning, with human plasma purified C1r and C1s. Four anti-C1s VHHs and 5 anti-C1r VHHs were chosen regarding their reactivity. The coding sequence were then optimized and cloned into a pET24+ plasmid with a pel B signal peptide and a C-terminal 6×His tag (Twist Bioscience, San Francisco, CA, USA). For this study, only two anti-C1r and 1 anti-C1s VHHs will be described as representative examples.

### 2.2. Expression and Purification of the VHHs

*E. coli* BL21 (DE3) (CMC0014, Sigma-Aldrich, St. Louis, MO, USA) were transformed with pET24+ coding for anti-C1r/C1s VHHs and grown in LB medium containing 50 µg/mL kanamycin at 37 °C under agitation. Large scale production was done in a TB medium supplemented by 50 µg/mL kanamycin, and the bacteria were induced with 1 mM IPTG when reaching 0.5–0.8 OD at 600 nm and incubated overnight at 28 °C. The culture was harvested by centrifugation at 3000× *g* for 20 min at 4 °C and the pellet was resuspended in Buffer A (50 mM Tris, 300 mM NaCl, 5 mM Imidazole, 5% (*m*/*v*) Glycerol, pH 8.0). The suspension was supplemented with 1 mM PMSF, 10 µg/mL DNAse, 250 µg/mL Lysozyme and 5 µg/mL MgSO_4_, and incubated under agitation at 4 °C. After sonication, the lysate was centrifuged at 15,000× *g* for 30 min at 4 °C to collect the supernatant.

The filtered supernatant was loaded on a HiTrap Chelating column (5 mL, 17040801, Cytiva, Marlborough, MA, USA) charged with NiCl_2_ and equilibrated with Buffer A at a flow rate of 2 mL/min. After a washing step with 10% Buffer B (Buffer A with 500 mM Imidazole; 10 CV), elution was carried on with 50% Buffer B (10 CV). The fractions containing the VHHs were pooled, concentrated (Amicon Ultra-15 mL-3 kDa, Merck KGaA, Darmstadt, Germany) and further polished by SEC on a HiLoad Superdex 16/600 75 pg column (Cytiva) equilibrated in 1X PBS pH 7.0 (ET330, Euromedex, Souffelweyersheim, France) at a flow rate of 1 mL/min.

### 2.3. Purification of C1r and C1s Proteases and Their Fragments

All the different proteins used in this study are described in Table 1 with their characteritics.

#### 2.3.1. Plasma C1r and C1s and Tetramer

Active C1r and C1s were purified from human plasma following the protocol published previously [26]. The two proteins were mixed in an equimolar ratio with calcium to reconstitute the (C1r)_2_/(C1s)_2_ tetramer [27], in order to provide the antigen needed for the lama immunization.

#### 2.3.2. Recombinant Versions of C1r and C1s Proteases and Fragments

Plasmids encoding recombinant C1r and C1s mutants/fragments, were obtained by site-directed mutagenesis (QuickChange II XL kit, Agilent Technologies, Santa Clara, CA, USA) of pcDNA3.1 C1s-WT Flag [12] or pcDNA3.1 C1r-WT Strep [11] and confirmed by DNA sequencing (Genewiz, Leipzig, Germany). The pcDNA3.1 encoding C1s CCP1-2 SP Strep and CEC CCP1 His were previously described [28]. rC1s CCP2 SP was expressed and purified as described previously [29,30].

For the Strep tagged proteins, Expi293F cells (Gibco, Thermo Fisher Scientific, Grand Island, NY, USA) were cultured in Expi293 Expression medium (Gibco) at 37 °C, 120 rpm, 8% CO_2_. Each plasmid construct was transfected in 100 mL cell culture with Expifectamine according to the manufacturer’s protocol. The culture supernatant was collected and submitted to two successive centrifugation steps (1500 rpm for 5 min and 4000 rpm for 30 min at room temperature) before dialysis against 100 mM Tris, 150mM NaCl, 1 mM EDTA, pH 8.0. After dialysis the filtered supernatant was loaded on a 2 mL Strep-Tactin Sepharose resin (IBA Lifesciences, Göttingen, Germany). The purification was done following the manufacturer’s protocol at a 10 mL/h flow rate.

For the C1r proteins the pooled fractions were dialyzed against 50 mM Tris, 150 mM NaCl, pH 7.4 (Tris/NaCl buffer). For the rC1s CCP1-2 SP Strep, purification was achieved with a supplementary SEC on a HiLoad Superdex 16/600 75 pg column (Cytiva), equilibrated in Tris/NaCl buffer at a 1 mL/min flow rate. The purified proteins were concentrated, up to 0.5 mg/mL, on an Amicon Ultra-4 mL-10 kDa (Merck).

#### 2.3.3. Proenzyme Versions of C1r and C1s Proteases and Tetramer

The mutation of the active site serine into alanine (SA) was used to produce the recombinant proenzyme version of these enzymes. A stable HEK293F cell line expressing the recombinant proenzyme tetramer (rC1r SA Strep)_2_/(rC1s SA Flag)_2_ was generated as explained in Bally et al. [31], by cotransfection of pcDNA3.1 C1r Strep (S637A) and pcDNA3.1 C1s Flag (S617A). The recombinant proenzyme tetramer was produced in Freestyle 293 medium and the cell culture supernatant harvested and centrifuged as described above.

The filtered supernatant was loaded on a 2 mL anti-Flag M2 affinity gel column (Sigma, A2220) equilibrated in 1X TBS (Euromedex, ET220) containing 2 mM CaCl_2_ (TBS-Ca). The unbound material was then washed with TBS-Ca buffer and the (rC1r SA Strep)_2_/(rC1s SA Flag)_2_ was eluted by addition of 6 mL (3CV) of Flag peptide diluted in the wash buffer at 100 µg/mL. The pooled fractions were dialyzed against wash buffer and further purified by SEC on a HiLoad Superose 6 pg 16/600 column equilibrated in TBS-Ca, at a flow rate of 1 mL/min. Finally, the tetramer was concentrated (Amicon Ultra-5 mL-30 kDa, Merck) at approximately 0.5 mg/mL.

rC1rSA and rC1sSA were obtained from proenzyme tetramer (rC1r SA Strep)_2_/(rC1s SA Flag)_2_. rC1r SA and rC1s SA were separated by 5 mM EDTA, incubated for 1 h at 4 °C and purified by Strep-Tactin chromatography as described above. rC1s SA Flag is collected in the flowthrough and bound rC1r SA Strep is eluted. Both proteins were dialyzed against Tris/NaCl buffer and concentrated (Amicon Ultra-4 mL-10 kDa, Merck).

### 2.4. Determination of VHHs Specificity Using ELISA

#### 2.4.1. Binding of VHHs to C1r and C1s Proteases by Direct ELISA

The different proteins/fragments were coated at the equivalent concentration of 5 µg/mL of C1r/C1s from plasma, corresponding to 57.8 pmol/mL for C1r and 62.5 pmol/mL for C1s, diluted in PBS, on a 96-well MaxiSorp Plate (439454, Nunc, Thermo Fisher Scientific, Roskilde, Denmark), overnight at 4 °C. Wells were washed three times with 300 µL of PBS-Tween 0.05% (*w*/*v*; PBS-T) and then blocked with 200 µL of PBS-BSA 2% (*w*/*v*; PBS-BSA) for 1 h at RT. The plate was washed as described above. A serial dilution of the VHHs, from 10 to 0.0001 µg/mL in PBS-T-BSA 0.5% (*w*/*v*; PBS-T-BSA), was added to the wells, and incubated for 2 h at RT. After a washing step, 100 µL of secondary anti-His antibody coupled to horseradish peroxidase (Sigma A7058; 1:50,000 dilution in PBS-T-BSA) were added and incubated 1 h at RT. A final wash step was performed and 100 µL of the substrate 3,3′,5,5′-tetramethylbenzidine (TMB, Merck CL07) was added and incubated 5 min in the dark. Reaction was stopped by addition of 100 µL of H_2_SO_4_ 1 N. Optical densities (OD) at 450 nm were read using a BMG plate reader. 

#### 2.4.2. Binding of C1sCl7 VHH to rCEC CCP1 by Sandwich ELISA

First, 96-well Maxisorp plates (Nunc, 439454) were coated with VHHs serial dilution from 10 to 0.0001 µg/mL in PBS, and incubated at 4 °C overnight. Wells were subsequently washed three times with 300 µL of PBS-T and then blocked with 200 µL of PBS-BSA during 1 h at RT. After three washes as described above, 100 µL of rC1s CEC CCP1 were added at a concentration of 62.5 pmol/mL in PBS-T-BSA and left for 2 h at RT. The wells were then washed, and in house rabbit polyclonal anti-C1s primary antibodies (100 µL, 1:4000 dilution in PBS-T-BSA) were added and kept for 2 h at RT. After a washing step, incubation for 1 h at RT was performed with 100 µL of anti-rabbit IgG secondary antibody coupled to HRP (Sigma A0545; 1:30,000 dilution in PBS-T-BSA). After a final wash, 100 µL of TMB was added for 10 min in the dark. The reaction was stopped by addition of 100 µL of H_2_SO_4_ 1 N prior to OD 450 nm measurement.

### 2.5. Western Blot Analyses

Proteins separated by SDS-PAGE were transferred on nitrocellulose membrane using a Trans-Blot Turbo RTA Mini 0.2 µm Nitrocellulose Transfer Kit (#1704270, Bio-Rad Laboratories, Hercules, CA, USA) and a Trans-Blot Turbo transfer system (Bio-Rad) according to the manufacturer’s protocol. The membrane was washed in PBS, then saturated with 5% milk (*w*/*v*) in TBS and incubated overnight at 4 °C with gentle agitation. The membrane was washed three times in TBS-Tween 0.05% (*w*/*v*; TBS-T). The VHHs were added at 1 µg/mL in TBS-T-BSA 1% (*w*/*v*; TBS-T-BSA), for 1 h at RT. After a washing step, HRP coupled anti-His antibodies (Sigma, A7058) were added (1:2000 dilution in TBS-T-BSA) for 2 h at RT. After a wash and equilibration in TBS, revelation was done using ECL kit (Cytiva-Amersham, 29013864) and the membranes were imaged with Bio-Rad Chemidoc.

### 2.6. Potential Inhibitory Effect of VHHs on C1s Cleavage by C1r

Here we wanted to check how pre-incubating the VHHs with C1r or C1s would impact the cleavage of proenzyme C1s by C1r. To follow the cleavage of proenzyme C1s, 10% molar ratio of C1r was added to rC1s SA (38.6 pmol), and incubated 2 h at 37 °C.

To check the impact of the VHHs on the cleavage, they were preincubated with their protein target (C1r or C1s) overnight at 4 °C under agitation. Anti-C1r VHHs were mixed with plasma C1r, at 2–1–0.5 molar ratios for 1 C1r in Tris/NaCl buffer. Alternatively, anti-C1s VHHs were incubated with 2 molar ratios of rC1s SA. C1r was then added to the C1s- anti-C1s VHH mixes at 2.5–5–10% molar ratio of C1s. All the mixes were incubated 2 h at 37 °C. The reaction was stopped by addition of reduced 4X Laemmli buffer, and incubated 10 min at 95 °C. The solutions were then analyzed by SDS-PAGE using Novex Tris Glycine 10–20% gels (Invitrogen, Thermo Fisher Scientific, Carlsbad, CA, USA).

### 2.7. Complex Formation Analysis by SEC

Formation of secondary and ternary complexes of VHHs with their target was studied by SEC. Mixes of VHHs, at a 2:1 molar ratio, with rC1r CCP2 SP Strep (100 µg) were prepared and incubated overnight under agitation at 4 °C. Controls were done in the same conditions. Samples were analyzed with a Superdex 200 10/300 gel column (Cytiva), equilibrated in TBS, at 0.5 mL/min.

### 2.8. Immunohistochemistry

Formalin fixed paraffin embedded (FFPE) human skin sections were processed for immunohistochemical detection of complement C1r and C1s using either conventional rabbit antibodies or anti-C1r and anti-C1s VHH single domain antibodies (nanobodies). Slides were dried for 30 min at 37 °C, then deparaffinized through three successive 2 min baths of Clearene, followed by a graded ethanol series (100%, 90%, 70%, 50%, 2 min each) and rehydrated in distilled water, then equilibrated in TBS.

Heat induced epitope retrieval was performed on a Dako PT Link (Dako, Glostrup, Denmark) using high pH EnVision FLEX Target Retrieval Solution, with a programmed cycle of 75 °C followed by 97 °C for 30 min and a return to 65 °C. Slides were rinsed in Dako Wash Buffer and equilibrated for 5 min in TBS. Sections were delimited with a hydrophobic barrier pen (Dako, Les Ullis, France). Endogenous peroxidase activity was quenched with 200 µL of 33% H_2_O_2_ in distilled water for 15 min, followed by a 2 min wash in TBS containing 0.04% Tween 20 (TBS T) under gentle agitation. Non-specific binding was blocked with Protein Block (Enzo Life Sciences, Lyon, France) for 30 min, with no rinse step before primary antibody incubation.

Primary antibodies and VHHs were applied at 200 µL per slide and incubated overnight at 4 °C. Conventional rabbit anti-C1r (Proteintech, Euromedex, 17346-1-AP) and anti-C1s (Proteintech, 14554-1-AP) antibodies were used at 1/200, with a matched rabbit isotype control. Anti-C1s VHH (C1sCl7, final concentration 5.6 µg/mL) with a non-target VHH (control, final concentration 5.6 µg/mL). Anti-C1r VHH (C1rCl1, final 9 µg/mL) with the non-target VHH (control, final 9 µg/mL), so that each nanobody was matched to its isotype control at an identical final concentration.

The next day, slides were washed twice for 2 min in TBS T under agitation. For VHH stained sections, a bridging step was added by incubating slides with MonoRab Rabbit Anti-Camelid VHH Cocktail (A02014, GenScript, CliniScience, Nanterre, France) at 1/500 (final 1 µg/mL) for 45 min, followed by two TBS T washes. All slides, conventional and VHH stained, were then revealed with undiluted Anti-Rabbit PolyView Plus HRP (Enzo) for 30 min, followed by two TBS T washes. Peroxidase activity was developed with DAB (Dako, 1 drop per mL of diluent), with development times empirically optimized for each target: 1 min 15 s for conventional anti-C1r, 5 min for conventional anti-C1s and rabbit isotype control, 5 min for anti-C1r VHH, and 9 to 10 min for anti-C1s VHH and the matched VHH control.

Sections were rinsed in TBS T for 2 min and in distilled water for 3 min, counterstained with Mayer’s hematoxylin (200 µL per slide) for 6 min, rinsed in distilled water for 2 min, and mounted with pre-warmed Glycergel (135 to 140 °C). Whole slides were digitized on a Hamamatsu NanoZoomer (Hamamatsu Photonics K.K., Hamamatsu, Japan).

### 2.9. SPR Analyses

SPR analyses were conducted at 25 °C on Biacore T200 instrument (Cytiva).

Anti-C1r VHHs were diluted to 5 µg/mL in MES pH 6.0 then immobilized on a CM5 Series S sensor chip (Cytiva) by standard amine coupling chemistry, at a density of 300 Response Units (RU). All the coupling steps without immobilized protein were done to realize a reference surface.

Multi-cycle kinetics analyses were done with TBS-Ca supplemented with 0.05% (*v*/*v*) Tween-20 as running buffer, at a flow rate of 30 µL/min. The analytes were injected at increasing concentrations from 0 to 32 nM in running buffer. The surfaces were regenerated by injection of 10 mM Glycine pH 3.0 for 30 s. The experiments were performed in duplicate.

Analysis of the sensorgrams was achieved using the Biacore T200 evaluation 3.1 software (Cytiva), by subtracting the signals of the reference surface and buffer blank (double referencing). A 1:1 binding model fitting was performed for plasma C1r, rC1r CCP1-2 SP Strep, and (rC1r SA Strep)_2_/(rC1s SA Flag)_2_ to determine the association and dissociation rate constants, as well as the resulting dissociation constants. For the rC1r CCP2 SP Strep fragment, the dissociation constants were calculated using a steady-state analysis, with the measured responses at equilibrium plotted versus the analyte concentrations. All the values are shown as the means and standard deviation of the duplicates.

### 2.10. Detection of C1r in Biological Fluids by Sandwich ELISA

A coating solution of C1rCl1 VHH, at 5 µg/mL in PBS, was added in the wells of a 96-well Maxisorp plate (Nunc, 439454), and incubated overnight at 4 °C. Three successive washes were performed with 300 µL of PBS-T. The wells were blocked with 200 µL of PBS-BSA for 1 h at RT. After a washing step, the samples diluted in PBS-T-BSA were added and incubated for 2 h at 37 °C. For biological fluids the dilutions tested are the following: 1:100 for serum (with addition of 5 mM EDTA), 1:100 for plasma EDTA, 1:100 for transfected cell culture supernatant and at 1 µg/mL for plasma purified C1r as a control. C1r depleted serum (A302, Complement Technology, Tyler, TX, USA) was used as negative control and treated as serum sample. Wells were washed and primary antibody, C1rCl6 with a mouse Fc tag (Nabgen), was diluted to 1 µg/mL in PBS-T-BSA and deposited in the wells, during 1 h at RT. After a washing step, the secondary antibody, anti-mouse IgG coupled to HRP (Sigma A9044; 1:40,000 dilution in PBS-T-BSA) was added and incubated during 1 h at RT. A final wash was done and 100 µL of TMB were incubated for 25 min in the dark. The reaction was then stopped by addition of H_2_SO_4_ 1 N and the OD 450 was read.

## 3. Results

### 3.1. Lama Immunization

For the initial step of lama immunization, purified human serum (C1r)_2_/(C1s)_2_ tetramer was chosen as the antigen. This choice allowed us to limit the number of injections to three into one lama, with the opportunity to get VHHs against both human C1r and C1s. The success of this immunization strategy was confirmed by ELISA, testing the sera against purified human serum C1r and C1s, at day 0 and day 54 using two dilutions (1/250 and 1/500).

### 3.2. Protein Production for Epitope Mapping

**Figure 1:**
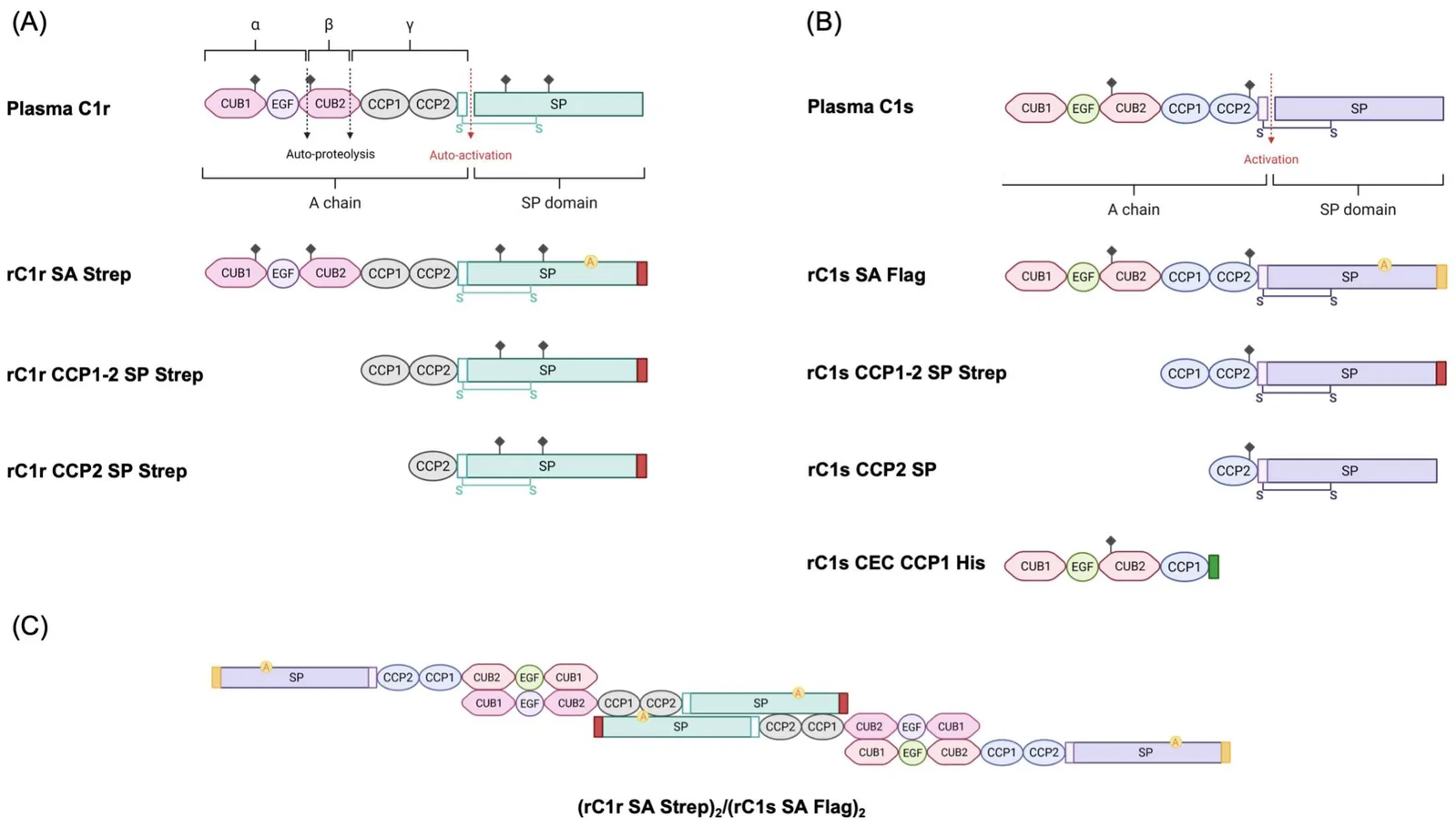
Schematic representation of the proteins and fragments used, highlighting their structural domains. (**A**) C1r and derivatives. Auto-proteolysis and auto-activation sites are shown with black and red arrows. Auto-proteolysis fragments, α, γ, β are represented, as well as A chain and SP domain. (**B**) C1s and derivatives. Activation site is symbolized with a red arrow, with the A chain and SP. (**C**) Proenzyme tetramer (rC1r SA Strep)_2_/(rC1s SA Flag)_2_. C-terminal tags are represented in red for Twin Strep tag, yellow for Flag tag and green for 6 × His tag. The active site serine mutations to alanine of the proenzyme forms are schematized as a yellow circled A. N-glycosylation sites are pictured as black diamonds.

To better characterize the part of the proteases recognized by the VHHs, a full set of fragments, including proenzyme or active forms, was prepared. All the proteins were successfully produced and purified as shown by SDS-PAGE analysis (Figure S1). The modular organization of the different proteins used in the study are shown in Figure 1. Plasma C1r is auto-activated, resulting in two bands corresponding to the A chain and SP domain in reducing SDS PAGE (Figure 1 and Figure S1). Auto-activated C1r is known to undergo auto-proteolysis leading to 3 N-terminal fragments (α, γ, β) that can be detected on the gel. Those fragments can also be seen with the shorter versions of C1r. In Figure 1B, plasma C1s is shown with its activation site that also results in the A chain and SP domain. Finally, the proenzyme tetramer is composed of the proenzyme version of the two proteases (Figure 1C).

### 3.3. VHH Purification and Initial Characterization (ELISAs, WB)

**Figure 2:**
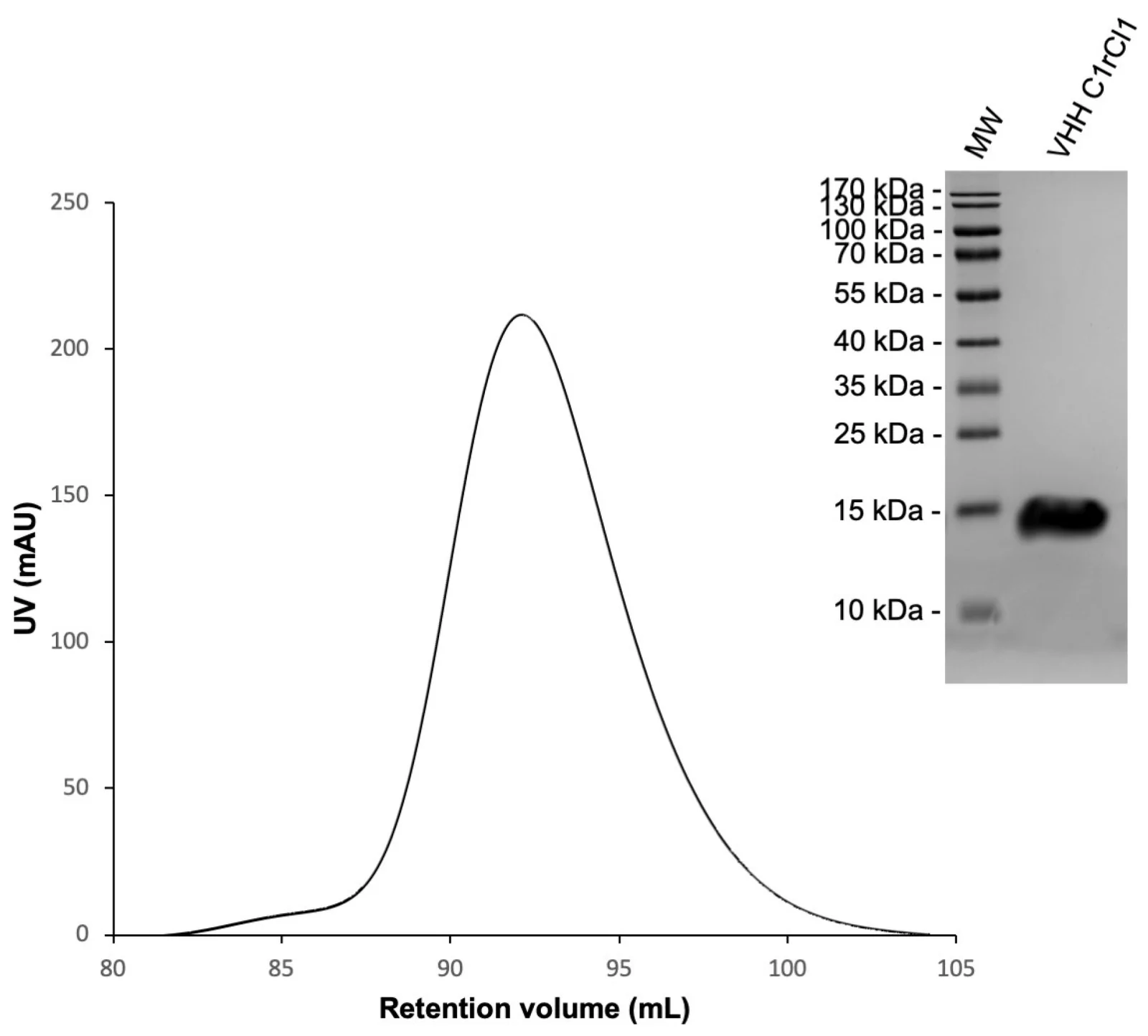
SEC and SDS-PAGE analysis confirm the purity of the C1rCl1 VHH. SEC profile of the purified VHH reveals a single peak at 92 mL, indicating a monomeric sample. SDS-PAGE analysis of the SEC pooled fractions shows a single band at ~15 kDa, consistent with the theoretical molecular weight of the VHH.

All the VHHs selected were efficiently produced, with a mean yield of 15 mg/L of culture, showing high purity, as exemplified for C1rCl1 VHH (Figure 2). ELISA were then performed to localize the protein area specifically targeted.

#### 3.3.1. Anti-C1r VHHs

**Figure 3:**
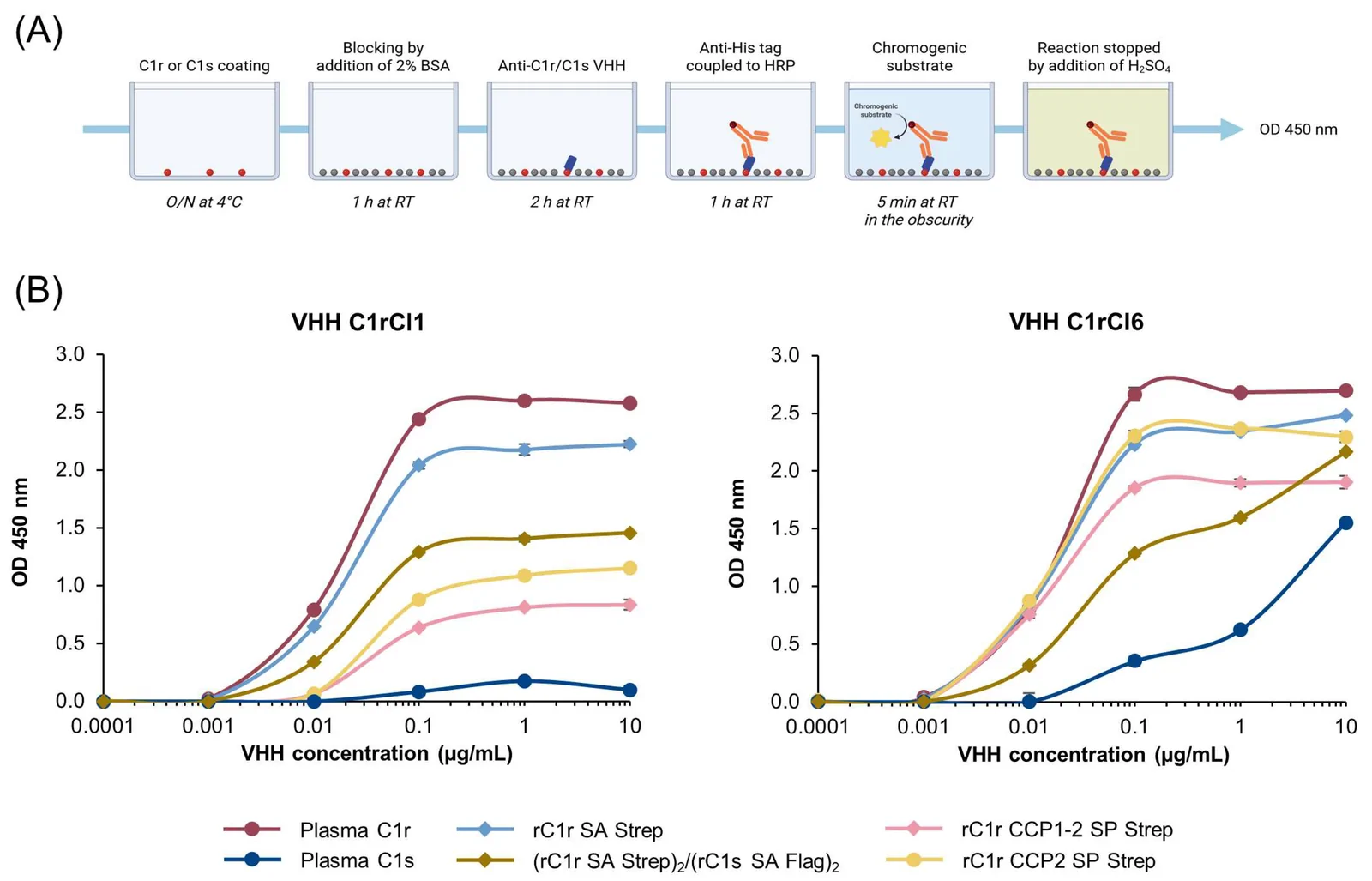
ELISA binding assay of C1rCl1 and C1rCl6 to different C1r and C1s targets. (**A**) Schematic representation of the direct ELISA workflow, as detailed in Section 2.4.1. (**B**) ELISA dose–response curves showing the optical density at 450 nm as a function of the VHH concentration (from 10 to 0.0001 µg/mL), C1rCl1 on the left and C1rCl6 on the right. Data are represented as means +/− SD (*n* = 2).

The C1rCl1 and C1rCl6 VHHs are recognizing all the C1r protein variants with no distinction between the fragments, the proenzyme (rC1r SA) and activated (plasma) forms. C1r Cl1 profile (Figure 3B, left) was similar to the other anti-C1r VHHs tested, except C1rCl6, the latter looking more as an exception (Figure 3B, left). Effectively, C1rCl6 showed atypical curves and differences in the maximum signal of the different proteins tested. Some cross-reaction with C1s was also exclusively observed for the C1rCl6 at high concentration, also seen with the tetramer.

Results of the Western blot show the interaction of C1rCl1 VHH with the full-length plasma C1r at ~100 kDa in non-reducing condition and with that of an auto-proteolytic fragment at ~15 kDa in reducing condition. This suggests a possible localization of the epitope on the CCP1-CCP2 fragment (also called γ; 19.6 kDa; [11]). With the rC1r CCP1-2 SP Strep fragment, a single band is detected in reducing condition, corresponding to the auto-proteolyzed CCP1-2 segment at ~15 kDa (also seen on Figure S1), confirming the precedent observation. For the C1rCl6 VHH, the observations are similar, but a cross reaction for C1s is seen in non-reduced conditions, as already observed by ELISA screening. The absence of detection of the rC1r CCP1-2 SP Strep fragment in non-reducing conditions is unexpected, and in fact a band can be observed at about 55 kDa in other WB experiments with C1rCl1, not with C1rCl6. This artifact, depending on the buffer used, could likely be influenced by a different epitope accessibility after binding of the recombinant fragment to the membrane. Nevertheless, the main interest resides in the detection of plasma C1r and its auto-proteolyzed fragments, which works properly in all conditions.

#### 3.3.2. Anti-C1s VHHs

**Figure 4:**
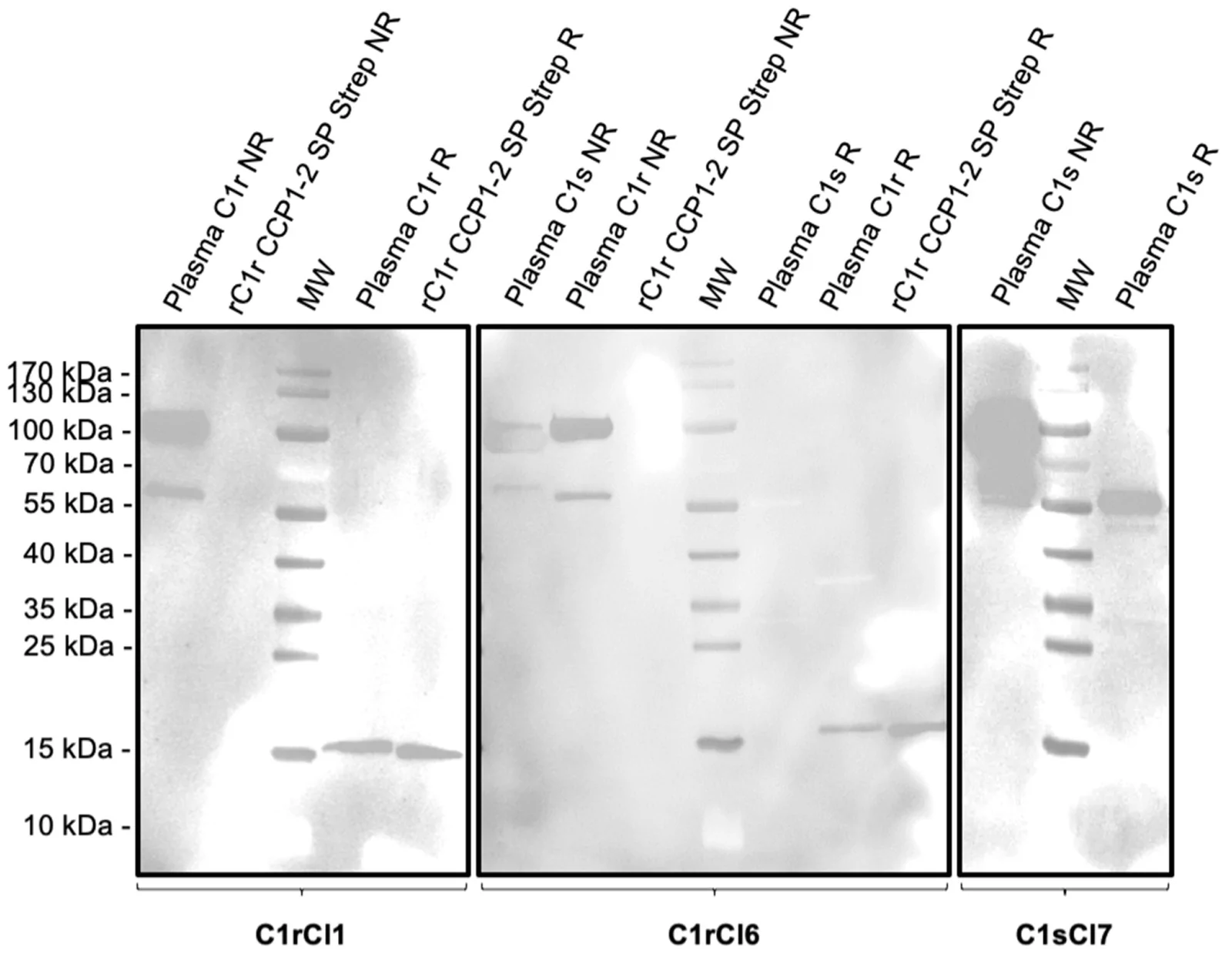
Western blot analysis of C1rCl1, C1rCl6 and C1sCl7 VHHs binding to their target. Proteins were separated by SDS-PAGE under reducing and non-reducing conditions, transferred to a nitrocellulose membrane, and probed with either C1rCl1, C1rCl6 or C1sCl7 VHH at 1 µg/mL. Binding was detected using HRP-conjugated anti-His antibody and chemiluminescence.

The C1sCl7 VHH specificity was also checked by WB and ELISAs. The WB shows C1s recognition in both reducing and non-reducing conditions (Figure 4, right). A specific recognition on the A chain containing CEC CCP1-2 modules is seen in reducing condition (around 60 kDa).

**Figure 5:**
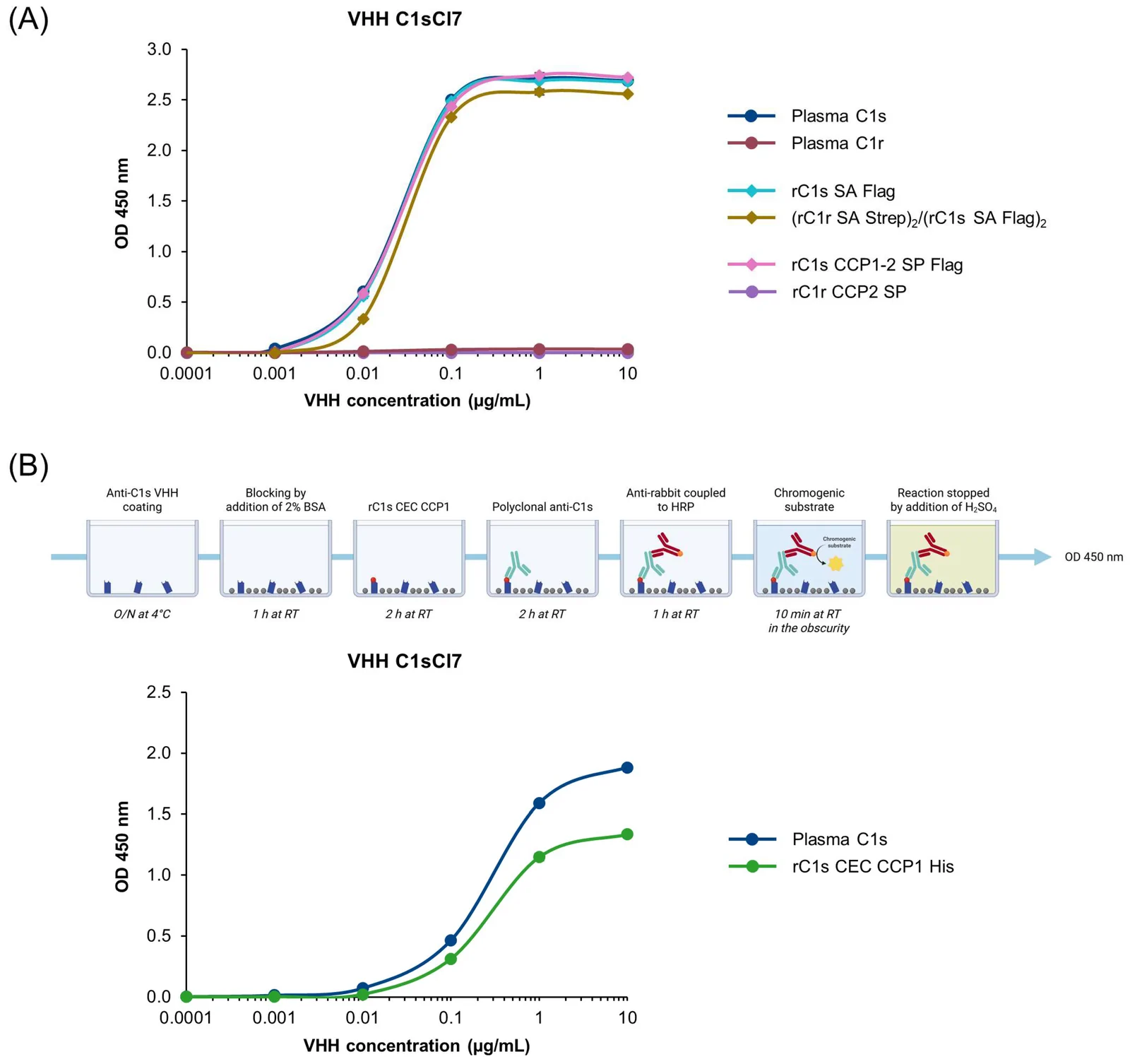
ELISA binding assay of C1sCl7 to different C1s and C1r targets and schematic representation of the test. (**A**) ELISA dose–response curves of the VHH C1sCl7 (from 10 to 0.0001 µg/mL), using the direct ELISA workflow shown on Figure 3. (**B**) Schematic representation of the sandwich ELISA workflow, as detailed in Section 2.4.2, with the corresponding ELISA dose–response curves of the VHH (from 10 to 0.0001 µg/mL). Data are represented as means +/− SD (*n* = 2).

The C1sCl7 VHH yielded similar signals in ELISA with the proenzyme rC1s SA, the active plasma C1s and the rC1s CCP1-2 SP fragment (Figure 5A). These results thus show that the VHH recognizes the C-terminal CCP1-2 SP fragment, without any distinction between the proenzyme and active forms. The shorter C-terminal CCP2 SP fragment was not recognized, indicating that the epitope might be on the CCP1 module. This result was confirmed with a sandwich ELISA (Figure 5B) using the rC1s CEC CCP1 His fragment, which showed interaction with the C1sCl7 VHH. Together, these observations suggest that the epitope for C1sCl7 could be localized on the CCP1 module.

Furthermore, no cross reaction with C1r was observed, neither with MASP-2, the homologous enzyme of the lectin complement pathway sharing the same cleavage specificity for C4 and C2 as C1s (Figure S2) [32].

### 3.4. Distinct Epitopes and Comparative Affinity Parameters of Anti-C1r VHHS

**Figure 6:**
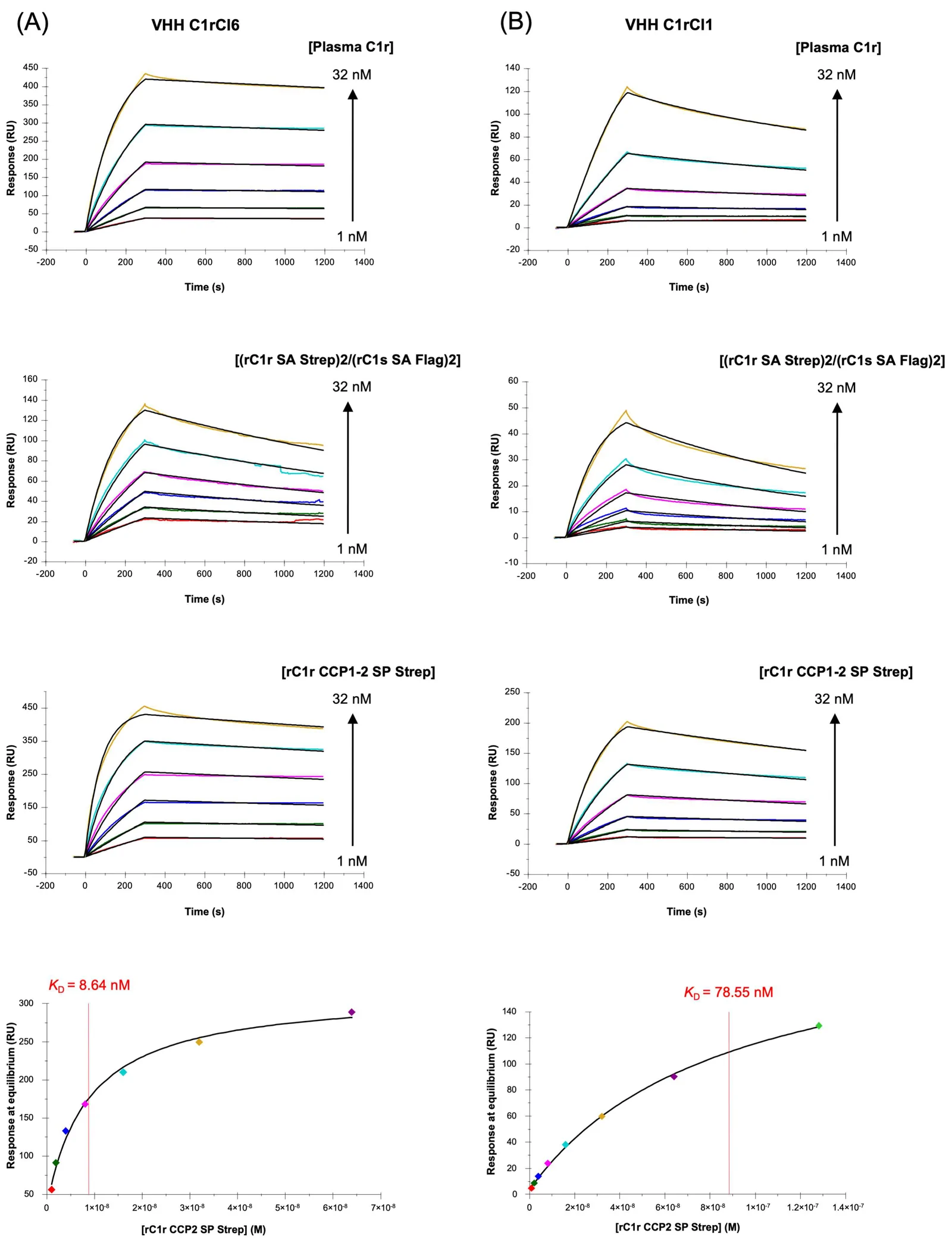
SPR analysis of binding interactions between C1rCl1 or C1rCl6 with their analytes. C1rCl1 (**A**) and C1rCl6 (**B**) were immobilized on CM5 series S sensor Chip. For plasma C1r, (rC1r SA Strep)_2_/(rC1s SA Flag)_2_, and rC1r CCP1-2 SP Strep, kinetic sensorgrams were recorded at concentrations from 0 to 32 nM, and data were fitted to a 1:1 binding model (with 0 nM subtraction). Each concentration is represented by a different color and the corresponding fitted curves are in black. For rC1r CCP2 SP Strep, steady-state binding was analyzed at concentrations from 0 to 64 nM for C1rCl6 and 0 to 128 nM for C1rCl1, and binding affinities (*K*_D_) are represented by vertical red lines. For each experiment, only one duplicate is shown. All the kinetic parameters and binding affinities are reported in Table 2.

Interaction was also analyzed by SPR experiments with anti-C1r VHHs immobilized on the chip, and injection of varying concentrations of different analytes (Figure 6). Kinetic analyses of the interactions between plasma C1r and rC1r CCP1-2 SP fragment yielded *K*_D_ values of the same order, comprised between 0.23 and 0.35 nM for C1rCl6 and 1.2–1.3 nM for C1rCl1, reflecting high affinities (Table 2 and Figure 6). We obtained only apparent *K*_D_ values for the tetramer by using a 1:1 fitting model, since it contains two C1r molecules and not only one. They show that the two VHHs have similar nanomolar affinity to the proenzyme tetramer.

The C1rCl6 VHH seems to have a better affinity than the C1rCl1 for most C1r analytes. Effectively, C1rCl6 has around four- to five-fold higher affinity than C1rCl1 for plasma C1r and the rC1r CCP1-2 SP Strep, respectively, explained mostly by a faster dissociation. Similarly, C1rCl6 VHH *K*_D_ is nearly 10-fold smaller for the rC1r CCP2 SP Strep fragment, (8.64 nM) compared to C1rCl1 VHH (78.5 nM) (Figure 6).

**Figure 7:**
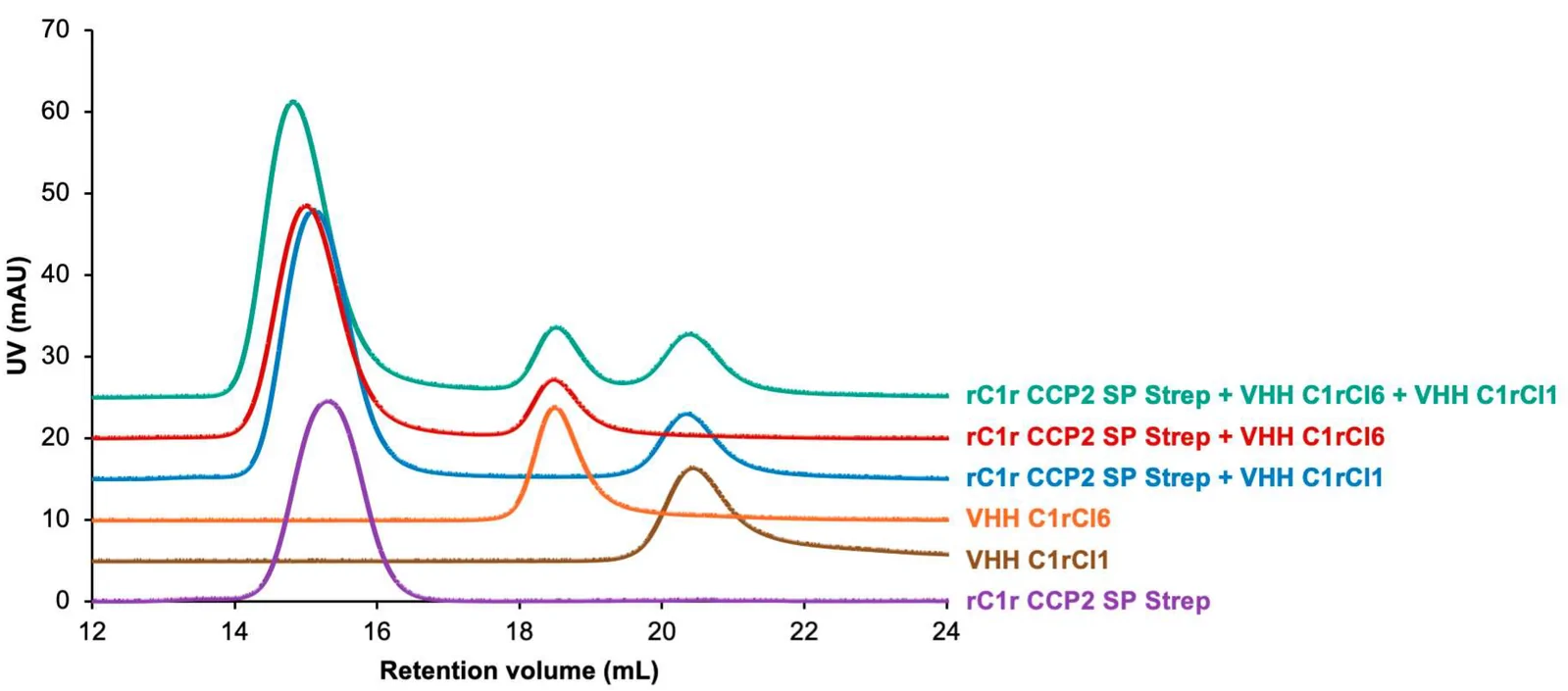
SEC analysis of complex formation between C1rCl1 VHH, C1r Cl6 VHH and rC1r CCP2 SP Strep fragment. SEC profiles of C1rCl1 alone, C1rCl6 alone, rC1r CCP2 SP Strep alone, binary complex of C1rCl1 and rC1r CCP2 SP Strep, binary complex of C1rCl6 and rC1r CCP2 SP Strep, and ternary complex of C1rCl1, C1rCl6 and rC1r CCP2 SP Strep. Chromatography was performed on a Superdex 200 10/300 gl column equilibrated in TBS.

Formation of secondary and ternary complexes between rC1r CCP2 SP Strep and the two anti-C1r VHHs was analyzed by SEC. Figure 7 shows that complexes between anti-C1r VHHs and rC1r CCP2 SP Strep are formed in solution. By mixing C1rCl1 and/or C1rCl6, a shift in retention volume (Table 3) is observed for the secondary and ternary complexes, as compared to the fragment or VHH alone, as well as a higher signal in mAU. This result highlights that these two anti-C1r VHHs bind different epitopes, and that they can bind together on the CCP2 SP portion of C1r. This result has also been confirmed by SPR experiments (Figure S3).

### 3.5. Potential Inhibitory Effect of the VHHs

**Figure 8:**
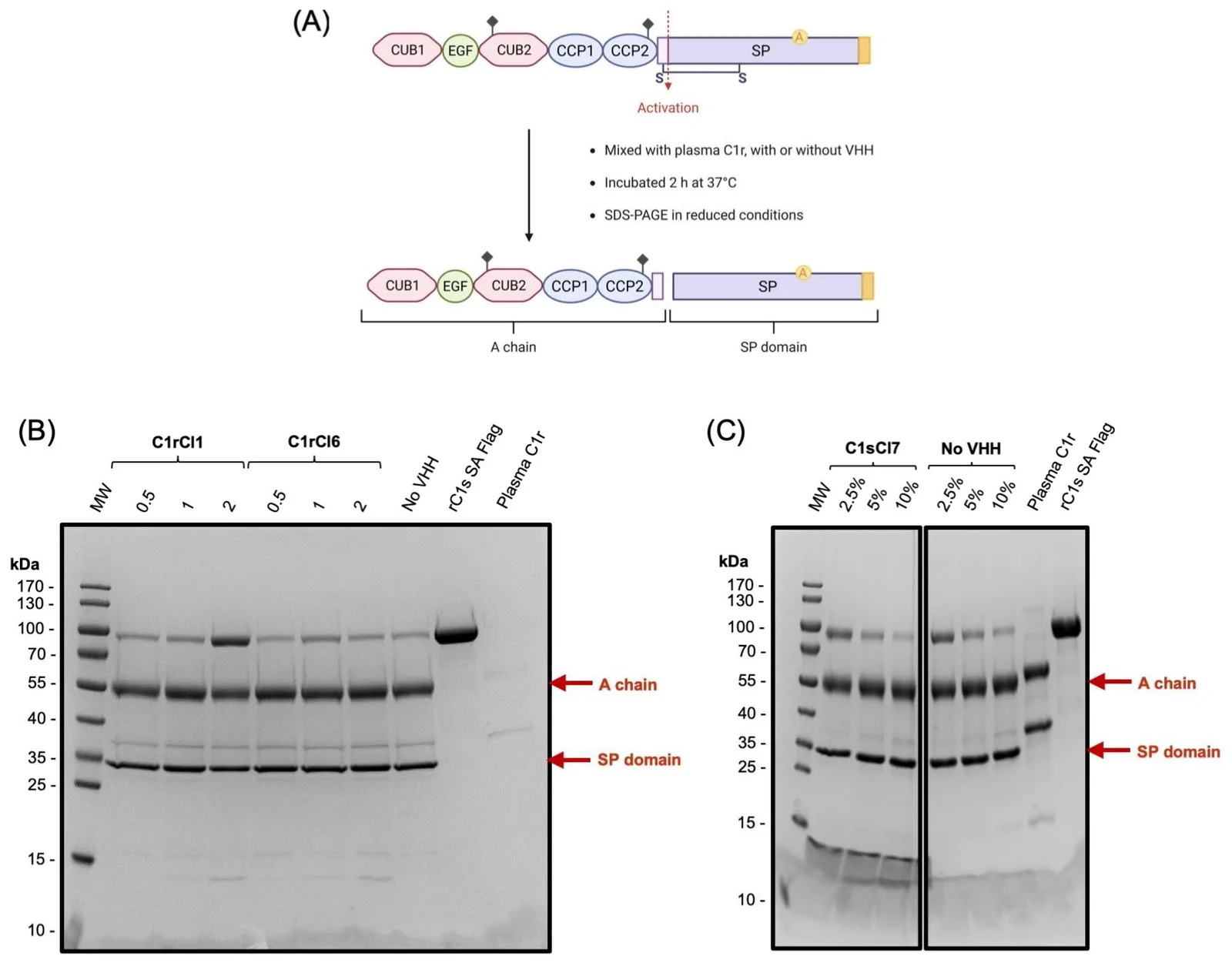
Effect of anti-C1r and anti-C1s VHHs on rC1s SA Flag cleavage by plasma C1r analyzed by SDS-PAGE. (**A**) Schematic representation of the cleavage. C-terminal Flag tag is represented in yellow. The active site serine mutation to alanine of the proenzyme form is schematized as a yellow circled A. N-glycosylation sites are pictured as black diamonds. (**B**) SDS-PAGE analysis of the rC1s SA cleavage by C1r with or without anti-C1r VHHs. Plasma C1r was used at 10% relative to rC1s SA Flag, with C1rCl1 or C1r Cl6 VHH added at ratios of 0.5, 1, or 2 VHHs per 1 C1r. (**C**) SDS-PAGE analysis of the rC1s SA cleavage by C1r with or without anti-C1s VHHs. Plasma C1r was tested at 2.5%, 5% or 10% relative to rC1s SA Flag, with C1sCl7 VHH added at a fixed ratio of 2 VHHs per 1 C1r. Controls without VHHs, and with the proteases alone are shown. Samples were separated on a 10–20% Tris-Glycine gel under reduced conditions and Coomassie blue stained.

The impact of the VHHs on the cleavage of the proenzyme rC1s SA proenzyme by plasma C1r was studied by pre-incubating the VHHs with either C1s or C1r (Figure 8A). As shown in Figure 8B, a decrease in the rC1s SA cleavage is observed when C1rCl1 VHH is added at a 2:1 molar ratio. This inhibition needs to be above a threshold level of VHH concentration, suggesting that all the sites of C1r must be saturated by the VHH in order to observe a decrease in the activating cleavage of rC1s SA. In contrast, the C1rCl6 (Figure 8B) and the C1sCl7 VHHs (Figure 8C) have no effect on the cleavage and show the same result as the control without VHH. The absence of effect for anti-C1s is fully consistent with the previous observation that this VHH targets the CCP1 module, and therefore should not interfere with the cleavage within the SP module.

### 3.6. Detection of C1r in Biological Fluids

**Figure 9:**
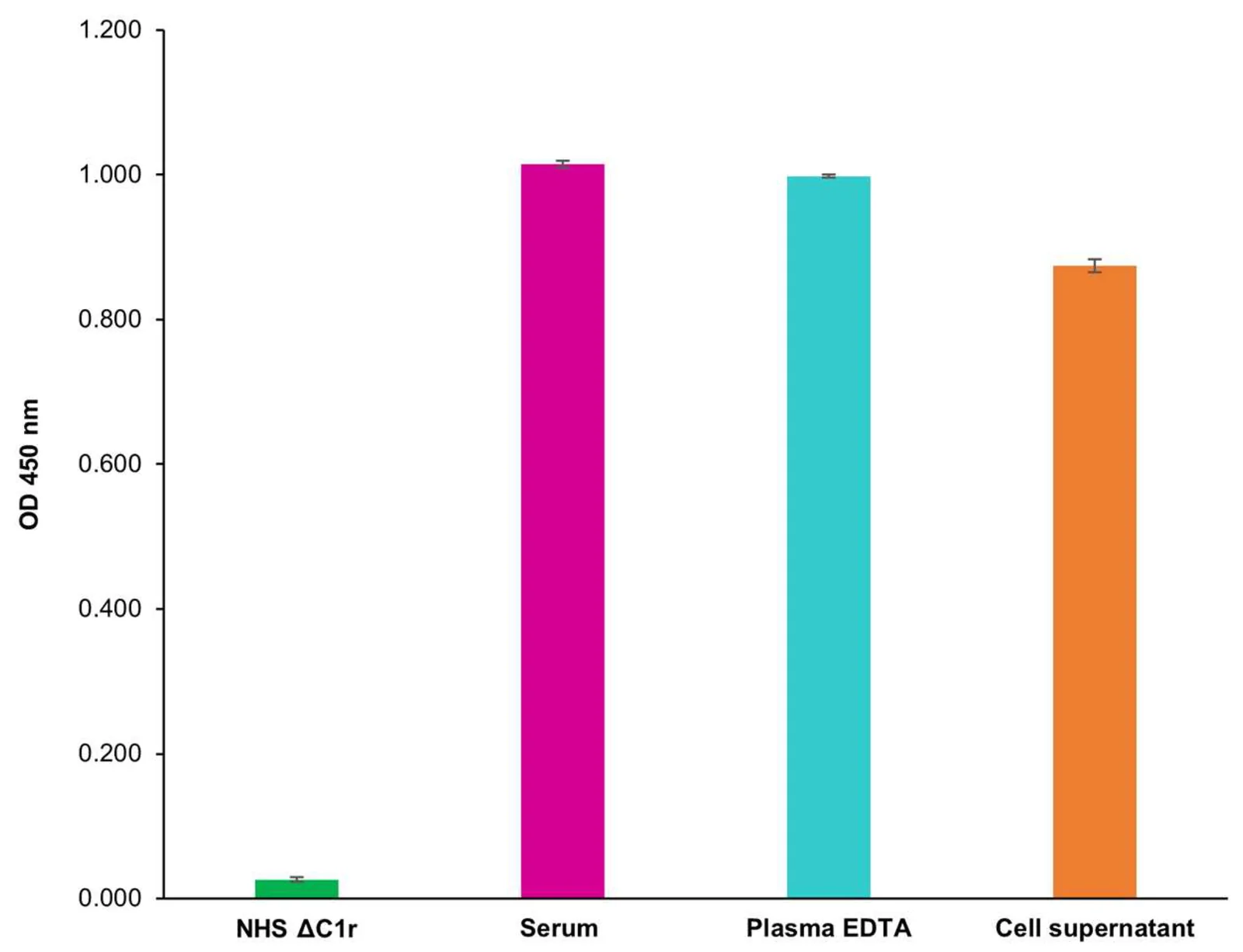
Detection of C1r in biological fluids using a sandwich ELISA. The use of the VHHs couple C1rCl1 and C1rCl6 fused to a Fc tag, as described in Section 2.10, was validated for the detection of C1r in different biological fluids at 1:100 dilution. Data are represented as means +/− SD (*n* = 2).

In order to check if the VHHs could be used to detect C1r in biological fluids, a sandwich ELISA was developed using C1rCl1 as a bait and C1rCl6 fused to a dimer Fc tag for the revelation step. As a first control, purified C1r was used for the initial setup. As shown in Figure 9, C1r can indeed be detected by this method at comparable levels in serum, plasma EDTA, the supernatant of Expi293F cells used to produce recombinant C1r, but not in serum depleted of C1r. Of note, the addition of 5 mM EDTA to serum was required to obtain the detection signal.

### 3.7. Application for Tissue Staining

**Figure 10:**
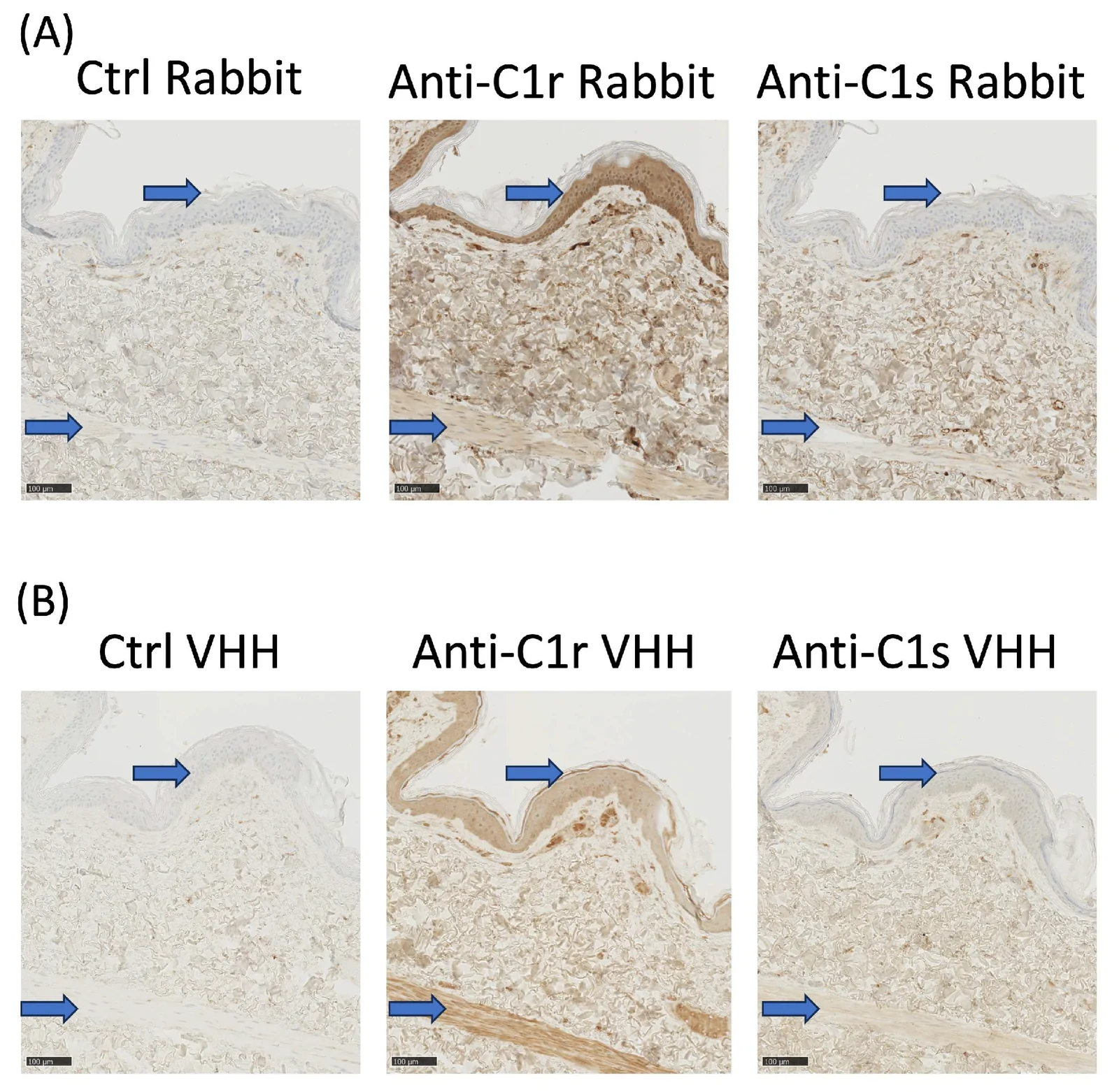
Strong staining for C1r and weak staining for C1s in fibroblasts and keratinocytes in human skin, detected with the rabbit anti-C1r and the VHH anti-C1r. (**A**) Staining with rabbit antibodies (brown): an isotype control (**left**), anti-C1r (**middle**) and anti-C1s (**right**). (**B**) Staining with the VHH: an irrelevant control (**left**), C1rCl1 anti-C1r (**middle**) and anti-C1s (**right**). Immunohistochemistry. Nuclear counterstaining with hematoxylin (blue). Scale bar at 100 µm. The upper blue arrow indicates the keratinocytes and the lower one—the fibroblasts.

In order to test the potential use of the anti-C1r and anti-C1s nanobodies for tissue staining, human skin sections were used for immunohistochemistry. The tissue staining was compared with the one obtained with commercial rabbit antibodies, as well as with controls (Figure 10). The staining obtained with the VHH is very clean and consistent with the one obtained with the rabbit antibodies. As underlined by the blue arrows in Figure 10, C1r is mostly seen in keratinocytes and fibroblasts, while the staining for C1s is weaker.

Strong positive staining is also observed for C1r in the sweat glands (Figure S4), but not for C1s. This shows that the two proteases locations are more dissociated in tissues than in the circulation, where they are associated as a tetramer bound to C1q inside the C1 complex.

## 4. Discussion

With the strategy of using the classical complement C1r2-C1s2 proteases tetramer for lama immunization, a set of anti-C1r and anti-C1s monodomain VHH antibodies have been developed and produced with high yields. Since C1r and C1s are homologous proteases sharing 36.7% of sequence identity, the possible drawback of this strategy was to possibly get some cross-reactivity with the two proteases, which happened with the C1rCl6 VHH, as illustrated in Figure 3.

Among the VHHs selected based on reactivity and sequence diversity, the best binders were prioritized for full characterization. This paper focuses on the three nanobodies that were validated for use with biological samples, as they represent the most informative and complementary profiles across the anti-C1r and anti-C1s specificities.

Epitope mapping localized the VHHs binding to the CCP modules of each protease. The two anti-C1r VHHs target distinct epitopes, enabling their use as a capture/detection pair for future C1r quantification in biological fluids, and one of them exerts a mild inhibitory effect on C1s activation. Proof-of-concept immunohistochemistry on human skin further demonstrated reliable tissue staining. These results establish a promising nanobody toolkit to study C1r and C1s in their emerging tissue and cell-intrinsic contexts.

In order to check if the VHHs could distinguish between the active and proenzyme forms of C1r and C1s, both active forms purified from serum and proenzyme forms recombinantly produced using SA mutations have been used. Using this mutation is indeed highly convenient to prevent any C1r or C1s auto-activation, and to obtain recombinant enzymes or fragments in a pure and stable proenzyme conformation. All VHHs similarly bound to both the active and proenzyme forms of C1r and C1s.

To better locate the protease region targeted by the VHHs, we used a set of recombinant fragments and several ELISA, WB and SPR binding analyses. These analyses have shown that the main epitopes are located near the CCP modules. This nicely fits with the previous observation that they cannot be used to distinguish whether the enzymes are in the active and proenzyme forms, since the conformational changes between these two forms is only located around the active site, within the SP domain. These epitopes are also accessible in the protease tetramer, as expected from the immunization protocol. More precisely, C1sCl7 targets the CCP1 module in C1s. Although the ELISA analyses suggest that C1rCl1 and C1rCl6 mainly target the CCP2 module, the SPR analyses show a better apparent affinity when the two CCP1 and CCP2 modules are present, and that the epitope is slightly less accessible within the protease tetramer.

We checked the absence of cross-reactivity with MASP-2 because its CCP modules play an important role in binding the complement C4 substrate, an interaction property shared with the CCP modules of C1s [33]. Although we are confident in the very high specificity of the VHH raised in this study, we should mention that CCP (also named sushi) modules are found in multiple copies in several complement proteins belonging to the Regulators of Complement Activation (RCA family), as well as in synaptic and neurological proteins included in the Sushi Domain (SUSD) Containing Family [34]. Checking possible cross-reactivity might be considered in the future for proteins thus possibly sharing closely related CCP epitopes. In the same line, the first VHH recognizing complement factor H CCP modules 18 to 20 was shown to target an epitope also recognized by anti-CFH autoantibodies and involved in atypical hemolytic uremic syndrome pathogenesis [35]. We would also be interested in checking whether the VVH raised in this study could compete with anti-C1s or anti-C1r autoantibodies [28].

We could detect potential inhibitory effect of C1rCl1 on C1s activation cleavage by C1r. Up to now, there are very few specific C1r inhibitors, with only the Borrelial pathogens proteins impacting the function of C1r as an escape mechanism, and involved in the Lyme disease [36]. Further protein engineering strategies might be used to increase the inhibitory potential of C1rCl1. Indeed, VHH are more and more used as molecular bricks that can be used in multiple copies covalently fused using small linkers and/or added to human IgG core domains such as IgG1 Fc [37,38]. VHH humanization and sequence optimization is often also required to achieve potential therapeutic use [38].

We tested the use of these VHHs in WBs, showing that they can be used both in reducing and non-reducing conditions for the full proteases (Figure 4), which is an important advantage, as the commercial polyclonal or monoclonal anti-C1r usually are not suitable for WB or recognize only the reduced form. In addition to this direct visualization tool, the fact that the protease interaction domain, including the N-terminal CUB1-EGF-CUB2 modules, remains free when bound to the VHH described here, could further open the way to co-immunoprecipitation experiments.

Although C1rCl6 showed the best binding for human C1r in initial ELISA screenings, as well as the best apparent affinity in SPR experiments, its partial cross-reactivity with C1s is a main drawback. This partial cross-reactivity could be explained by the fact that it recognizes one major and one minor epitope, the second one being shared by C1s. This would also explain its strongest apparent affinity. The fact that C1rCl6 and C1rCl1 could bind to distinct epitopes, forming ternary complexes with CCP2-SP, as shown by SEC analyses (Figure 7), and confirmed by SPR experiments (Figure S3), led us to the idea to use this VHH in fusion to Fc for the final revelation step of the sandwich ELISA able to detect C1r in fluids. The addition of 5 mM EDTA is required for the detection of C1r in serum, which suggests that a dissociation step is needed in this biological context where Ca^2+^ is present and the C1r is nearly entirely associated with C1s and C1q to form the C1 complex [2,39].

Finally, we also tested skin staining with C1rCl1 and C1sCl7, as a first proof-of-concept step towards a larger use of these VHHs for tissue staining. Whereas C1r and C1s are mainly found within the C1 complex in the circulation, skin staining here evidenced that these two proteases are not always present together in tissues, using both VHH and polyclonal antibodies. This is in agreement with previous findings in breast cancer, where C1r and C1s were not always expressed by the same tumor cells, demonstrated at protein and mRNA level in cell lines, in scRNAseq datasets of patient tumors and in situ, using RNAscope and SeqIF hyperplex immunofluorescence tissue imaging [24]. Our observations here, even if only with chromogenic staining, suggest intracellular staining pattern with both VHH and polyclonal antibodies, in agreement with our previous observations in breast [24] and renal cancer [17]. This is a relatively new area of research and requires further investigations. The newly generated VHH tools could be useful in this respect to better decipher the intracellular localization and function of C1r and C1s within tissues.

These first anti-C1r and anti-C1s nanobodies provide a promising toolkit to study these proteases in their classical circulatory context and within tissues and cells. Their high affinity, specificity, and compatibility with sandwich ELISA and tissue immunohistochemistry make them immediately applicable as research tools. These nanobodies could further support future patient follow-up and tissue analysis in diseases where C1r or C1s play a demonstrated pathological role, including systemic lupus erythematosus, pEDS and different types of cancer.

## Figures and Tables

**Table 1:** Protein characteristics used for quantification.

Proteins	Molecular Weight (Da)	Abs_280_ 0.1%, 1 cm
VHH C1rCl1	14,591	1.948
VHH C1rCl6	13,391	1.387
VHH C1sCl7	13,427	1.263
Human plasma C1r	86,500	1.24
Human plasma C1s	80,000	1.45
rC1r SA Strep (S637A)	81,133	1.407
rC1r CCP1-2 SP Strep	48,240	1.696
rC1r CCP2 SP Strep	40,381	1.779
rC1s SA Flag (S617A)	75,866	1.498
rC1s CCP1-2 SP Strep	46,885	1.884
rC1s CCP2 SP	36,461	1.640
rC1s CEC CCP1 His	39,452	1.237

**Table 2:** Kinetic and binding parameters determined from SPR experiments.

Immobilized VHH	Analyte	k_a_ (1/Ms)	k_d_ (1/s)	*K*_D_ (M)	R_max_ (RU)	Chi^2^ (RU^2^)
C1rCl6	Plasma C1r	2.17 ± 0.01 × 10^5^	7.63 ± 1.61 × 10^−5^	3.51 ± 0.72 × 10^−10^	518.70 ± 52.50	10.09 ± 3.16
	(rC1r SA Strep)_2_/(rC1s SA Flag)_2_	1.82 ± 0.39 × 10^5^	4.04 ± 0.24 × 10^−4^	2.26 ± 0.35 × 10^−9^	269.34 ± 118.94	2.25 ± 1.34
	rC1r CCP1-2 SP Strep	4.18 ± 0.36 × 10^5^	9.78 ± 0.66 × 10^−5^	2.36 ± 0.36 × 10^−10^	457.61 ± 44.39	31.21 ± 3.61
	rC1r CCP2 SP Strep *	-	-	8.64 ± 0.07 × 10^−9^	279.91 ± 3.60	73.64 ± 4.59
C1rCl1	Plasma C1r	6.50 ± 0.51 × 10^5^	8.77 ± 0.82 × 10^−4^	1.36 ± 0.23 × 10^−9^	148.45 ± 75.85	0.64 ± 0.08
	(rC1r SA Strep)_2_/(rC1s SA Flag)_2_	2.54 ± 0.22 × 10^5^	7.49 ± 0.66 × 10^−4^	2.95 ± 0.01 × 10^−9^	59.92 ± 16.32	0.75 ± 0.03
	rC1r CCP1-2 SP Strep	3.08 ± 0.99 × 10^5^	3.87 ± 1.67 × 10^−4^	1.23 ± 0.15 × 10^−9^	195.23 ± 28.33	2.32 ± 0.57
	rC1r CCP2 SP Strep *	-	-	7.85 ± 1.38 × 10^−8^	176.23 ± 47.80	1.92 ± 2.18

* For rC1r CCP2 SP Strep, steady-state analysis was used to obtain dissociation constants.

**Table 3:** Elution volume of the secondary and ternary complexes, compared to single proteins.

Proteins or Complexes	Elution Volume (mL)
rC1r CCP2 SP Strep + VHH C1rCl6 + VHH C1rCl1	14.81
rC1r CCP2 SP Strep + VHH C1rCl6	15.00
rC1r CCP2 SP Strep + VHH C1rCl1	15.10
VHH C1rCl6	18.48
VHH C1rCl1	20.43
rC1r CCP2 SP Strep	15.30

## Data Availability

The original contributions presented in this study are included in the article/Supplementary Material. Further inquiries can be directed to the corresponding author.

## Supplementary Materials

The following supporting information can be downloaded at: https://www.mdpi.com/article/10.3390/antib15050092/s1, Figure S1: SDS-PAGE analysis of the C1r and C1s proteins and fragments used; Figure S2: ELISA binding assay of C1sCl7 to rMASP-2 SA and SDS-PAGE of rMASP-2 SA; Figure S3: C1rCl1 and C1rCl6 VHHs can bind simultaneously on C1r in SPR binding analysis; Figure S4: Positive staining for C1r in sweat gland, detected with the rabbit anti-C1r and the VHH anti-C1r.

## Abbreviations

The following abbreviations are used in this manuscript:

pEDS	periodontal Ehlers-Danlos Syndrome
SA	mutation of the active serine into alanine (SA)
WB	Western blot
VHH	monodomain VHH antibodies obtained from camelid immunization
