# Supplementary material for: Targeting the Complement C1 Proteases Using Camelid-Derived Single Domain Antibodies: Production, Characterization and Possible Application for Tissue/Fluid Analysis
Source: Antibodies (Basel). 2026 Oct 5;15(5):92. doi: 10.3390/antib15050092 (PMC13700691; doi:10.3390/antib15050092)
Supplement: Supplementary file 1 [file antibodies-15-00092-s001.zip › antibodies-4429632-supplementary.pdf]

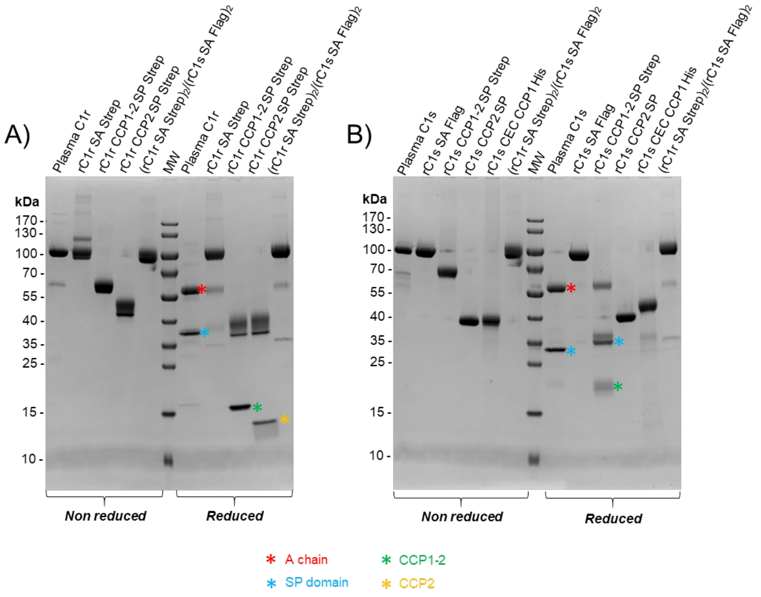

Figure S1: SDS-PAGE analysis of the C1r and C1s proteins and fragments used. Coomassie blue-stained SDS-PAGE gels, in reduced and non-reduced conditions, showing the electrophoretic profiles of plasma proteins and their recombinant versions, mutated or fragmented (A: C1r proteins; B: C1s proteins). Molecular weight markers (MW) in kDa are indicated on the left of each gel. For plasma C1r and C1s, a single band is visible in non reduced conditions and the two resulting bands of reducing conditions (A chain and SP domain) are also seen. Proenzyme forms are characterized by a major band in both conditions. For the fragments, a single band is observed in non reduced conditions, but multiple bands are visible in reduced conditions, resulting from proteolysis and/or activation. For the tetramer, a single band is observed as proenzyme C1r and C1s bands are superimposed.

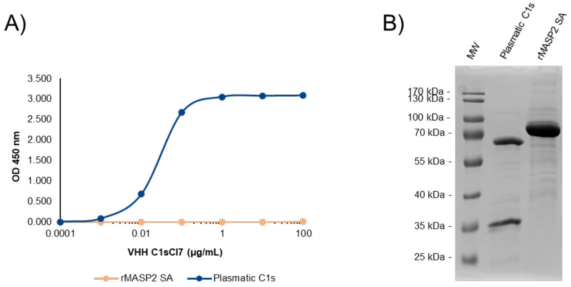

Figure S2: ELISA binding assay of C1sCl7 to rMASP-2 SA and SDS-PAGE of rMASP-2 SA. A) ELISA dose-response curves showing the optical density at 450 nm as a function of the VHH C1sCl7 concentration (from 10 to 0.0001 µg/mL). Data are corrected with the blank signal and represented as means +/- SD (n=2). B) Control of the rMASP-2 SA protein and plasma C1s by SDS-PAGE analysis in reduced conditions.

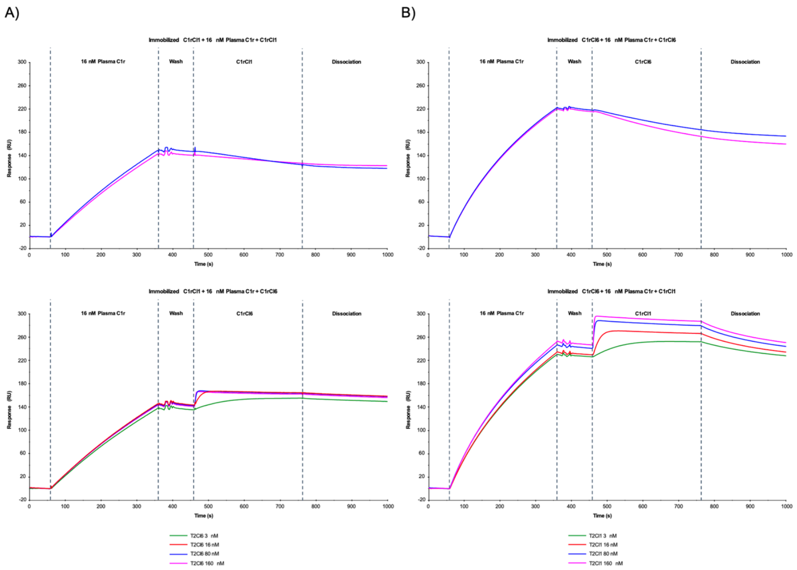

Figure S3: C1rCl1 and C1rCl6 VHHs can bind simultaneously on C1r in SPR binding analysis. C1rCl1 (A) and C1rCl6 (B) were immobilized on CM5 series S sensor Chip. After a first association with 16 nM plasma C1r, C1rCl6 or C1rCl1 were injected at different concentrations (3 to 160 nM). After injection of C1rCl1 on the chip with the same VHH immobilized, no binding is observed, and this absence of binding is also observed for the C1rCl6 in the same configuration. However, when injecting C1rCl1 on the chip with C1rCl6 immobilized, binding is observed, and this binding is equally observed for the couple in the other configuration. Curves were adjusted on the zero baseline.

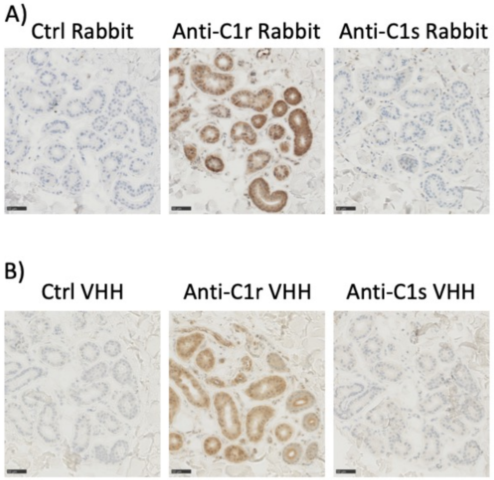

Figure S4: Positive staining for C1r in sweat gland, detected with the rabbit anti-C1r and the VHH anti-C1r. A) Staining with rabbit antibodies (brown): an isotype control (left), anti-C1r (middle) and anti-C1s (right) B) Staining with the VHH: an irrelevant control (left), anti-C1r (middle) and anti-C1s (right). Immunohistochemistry. Nuclear counterstaining with hematoxylin (blue). Scale bar at 50 µm.
